# Behavioral, Physiological, and Molecular Mechanisms Underlying the Adaptation of *Helicoverpa armigera* to the Fruits of a Marginal Host: Walnut (*Juglans regia*)

**DOI:** 10.3390/plants13192761

**Published:** 2024-10-01

**Authors:** Haiqiang Li, Xinzheng Huang, Long Yang, Haining Liu, Bing Liu, Yanhui Lu

**Affiliations:** 1 State Key Laboratory for Biology of Plant Diseases and Insect Pests, Institute of Plant Protection, Chinese Academy of Agricultural Sciences, Beijing 100193, China; jacky81611@163.com (H.L.); yanglong@caas.cn (L.Y.); liubing1945@126.com (B.L.); 2 Scientific Observing Experimental Station of Crop Pest in Korla, Key Laboratory of Integrated Pest Management on Crop in Northwestern Oasis, Ministry of Agriculture and Rural Affairs, Institute of Plant Protection, Xinjiang Academy of Agricultural Sciences, Urumqi 830091, China; 3 College of Plant Protection, China Agricultural University, Beijing 100193, China; huangxinzheng@cau.edu.cn

**Keywords:** *Helicoverpa armigera*, walnut fruit, adaptive mechanism, cytochrome P450, transcriptome

## Abstract

In northwest China, changes in cultivation patterns and the scarcity of preferred hosts have forced *Helicoverpa armigera* to feed on the marginal host walnut (*Juglans regia*). However, the mechanisms allowing this adaptation remain poorly understood. Here, we investigated the behavioral, physiological, and molecular mechanisms underlying the local adaptation of this pest to walnut fruits. The green husk and shell generally contained higher levels of phytochemicals than the kernel. Bioassays revealed that the phytochemical-rich green husk and shell were less preferred, reduced larval fitness and growth, and elevated the activity of detoxification enzymes compared to the nutrient-rich kernel, which were further supported by a larger number of upregulated detoxification genes in insects fed green husks or shells based on transcriptome sequencing. Together, these data suggest that P450 genes (*LOC110371778*) may be crucial to *H. armigera* adaptation to the phytochemicals of walnuts. Our findings provide significant insight into the adaptation of *H. armigera* to walnut, an alternative host of lower quality. Meanwhile, our study provides a theoretical basis for managing resistance to *H. armigera* larvae in walnut trees and is instrumental in developing comprehensive integrated pest management strategies for this pest in walnut orchards and other agricultural systems.

## 1. Introduction

The cotton bollworm, *Helicoverpa armigera* (Lepidoptera: Noctuidae), is a polyphagous agricultural pest that feeds on a wide range of host plants, including cotton, chickpea, tomato, tobacco, corn, sesame, hemp, sunflower, peanut, okra, and soybean [1,2,3,4,5,6], causing serious economic losses to agriculture worldwide [7,8]. Generalist herbivores such as *H. armigera* must continually shift to new, alternative, and nutritionally challenging host plants as the availability or quality of other hosts declines. In China, *H. armigera* has been reported to shift onto some marginal hosts when preferred host plants become scarce either due to seasonality or changes in cultivation or land use patterns. The increased cultivation of high-value horticultural crops in areas formerly dominated by cotton production has led to greater exposure of some marginal hosts to *H. armigera* feeding [7]. The Southern Xinjiang Uygur Autonomous Region, China, has an arid or semi-arid climate that is ideal for cultivating high-quality fruit of various species, including walnut (*Juglans regia*), jujube (*Ziziphus jujube*), apple (*Malus pumila*), grape (*Vitis vinifera*), pear (*Pyrus sinkiangensis*), and apricot (*Prunus armeniaca*). Recent changes in agricultural production in the region and advancements in farming technology have led to an increased level of exposure of these fruits to *H. armigera*. For example, since 2017, damage to walnuts by first-generation *H. armigera* has been reported in Aksu, Southern Xinjiang, resulting in significant economic losses for the local walnut industry [9,10]. However, the mechanisms underlying the local adaptation of *H. armigera* larvae to walnut fruits are unclear.

Polyphagous generalists such as *H. armigera* feed on a wide range of host plants [11,12,13], and in doing so their larvae encounter a wide range of plant allelochemicals, against which herbivorous insects have developed various mechanisms to overcome plant defenses [14,15,16], including behavioral, physiological, and transcriptional changes that promote better detoxification of defense compounds [17,18,19,20,21,22]. The metabolic detoxification of such defensive compounds is commonly categorized into three phases [23,24]. In Phase I, enzymes such as cytochrome P450 monooxygenases and carboxylesterases (CarE) act upon the toxin molecule, enhancing its hydrophilicity and the reactivity of its functional groups. In the second stage, Phase II enzymes, including UDP-glycosyltransferases (UGTs) and glutathione S-transferases (GSTs), facilitate the conjugation of endogenous molecules with toxins. Also, other transferases such as methyltransferases, acetyltransferases, phosphotransferases, and sulfotransferases contribute to this conjugation process [25,26]. The resulting conjugates are more hydrophilic and less reactive, diminishing their ability to pass through membranes. Finally, in Phase III, ATP-binding cassette (ABC) transporters bolster the transmembrane transport of toxins, thereby aiding their excretion. Many studies have demonstrated that in *H. armigera*, key detoxification enzymes, including cytochromes P450 (P450s), are involved in the detoxification of phytochemicals, helping this pest to adapt to diverse toxins produced by host plants [24,27]. For example, *CYP6AE14* is involved in the adaptation of *H. armigera* to gossypol, and *CYP303A1* plays a role in the metabolism of the plant allelochemical 2-tridecanone [27].

In recent years, with the rapid development of next-generation sequencing technology, an increasing number of studies have employed RNA-seq to explore adaptive changes in insects in response to different host plants or xenobiotic phytochemicals [28,29]. For example, transcriptomic analysis was used to determine the mechanism of adaptation for the maize pest *Diabrotica virgifera* to different host plants. This study found that the larvae of this rootworm underwent fewer transcriptional changes when feeding on an alternate host plant than when feeding on a marginal or poor host [30]. Transcriptome profiling of the larvae of the weevil *Pagiophloeus tsushimanus* exposed to the plant-derived volatile compound linalool showed that linalool exposure induced the expression of genes encoding for P450s, cuticular proteins, antioxidases, heat shock proteins, and digestive enzymes [23]. Moreover, a review of studies that used transcriptomic analyses to investigate how genome-wide transcriptional changes contributed to adaptation over time to novel or poor-quality host plants found that during that process [31], many genes involved in detoxification (P450s, CarE, UGTs, GSTs, ABC transporters), digestion, chemosensory perception, and transcription factors were dynamically expressed. For instance, transcriptome profiling of two tobacco-adapted species of the whiteflies *Trialeurodes vaporariorum* and *Bemisia tabaci* found that *CYP6DP1* and *CYP6DP2* (in *T. vaporariorum*) and *CYP6DP3* (in *B. tabaci*) played important roles in strong induced tolerance to the toxic allelochemical nicotine [32].

In the present study, we investigated for the first time the effects of walnut fruits (green husk, shell, or kernel parts) on the feeding preferences and fitness costs of *H. armigera*. Then, the detoxification activities of P450 and carboxylesterase enzymes in *H. armigera* larvae fed on the above three parts of walnut fruits were also assessed. Finally, the molecular mechanisms underlying the adaptation and detoxification of *H. armigera* larvae to these three walnut fruit parts were examined using RNA-seq and confirmed by qPCR analysis. This study was designed to develop novel strategies for the sustainable management of *H. armigera* in walnut orchards and improve understanding of the interactions between polyphagous pests and their host plants.

## 2. Results

### 2.1. Secondary Metabolite Content in Walnut Fruit

**Figure 1:**
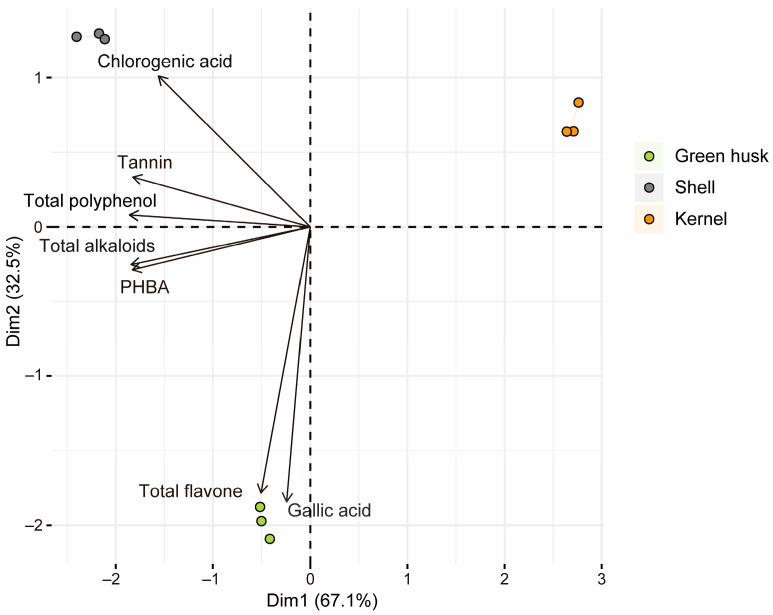
Biplot of principal component analysis (PCA) demonstrating the variation in seven secondary metabolites in the green husk, shell, and kernel of walnut fruit. The sample points with different colors indicate different parts of walnut fruit—the green husk (green), shell (gray), and kernel (orange), respectively. Solid lines with arrows represent the secondary metabolites; the cosine of the angle between them and the PC axis indicates the correlation between them on the PC axes (a smaller angle indicates a higher correlation).

Principal component analysis (PCA) indicated the variation in secondary metabolite content in different walnut fruit tissues. The first two principal components explained 67.1% and 32.5% of the variance, respectively, with a 99.6% cumulative percentage of explained total variances. Tannin, total polyphenol, total alkaloids, and PHBA had high contributions to the PC1 axis, whereas gallic acid and total flavone had high contributions to the PC2 axis. For the three parts of walnut fruit (green husk, shell, and kernel), the contents of tannin, total polyphenols, and chlorogenic acid were highest in the shell, while the contents of total flavones and gallic acid were highest in the green husk. Generally, the green husk and shell contained higher levels of phytochemicals compared to the kernel (Figure 1, Table S1).

### 2.2. Feeding Preferences of Larvae on Walnut Fruit Parts

**Figure 2:**
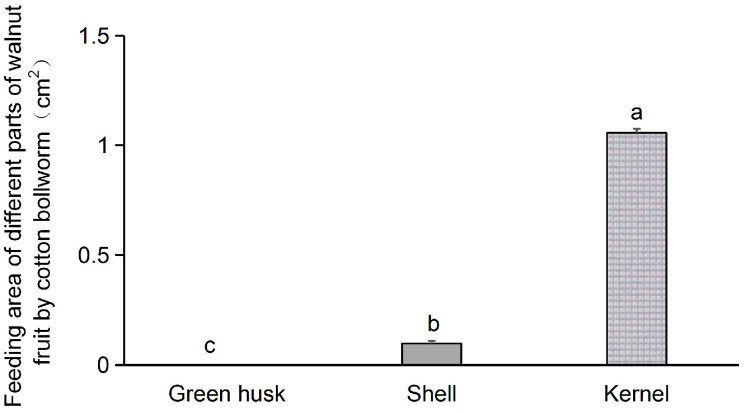
Feeding preferences of *Helicoverpa armigera* on different parts of walnut fruits (green husk, shell, kernel) at 24 h. The different letters indicate significant differences analyzed by one-way ANOVA followed by Tukey’s tests, *p* < 0.05.

The *H. armigera* larvae demonstrated a strong preference for the kernel over the green husk or shell of the walnut fruit. At 24 h after the onset of feeding, the consumed area of the kernel was 1.06 cm^2^ compared to only 0.10 cm^2^ and 0 cm^2^ for the shell and green husk. Consumption was significantly affected by food (*F* = 1259.0; *df* = 2, 6; *p* < 0.001) and was significantly greater in kernel-fed larvae than in shell- or green husk-fed ones (Figure 2).

### 2.3. Development of Larvae on Walnut Fruit Parts

**Figure 3:**
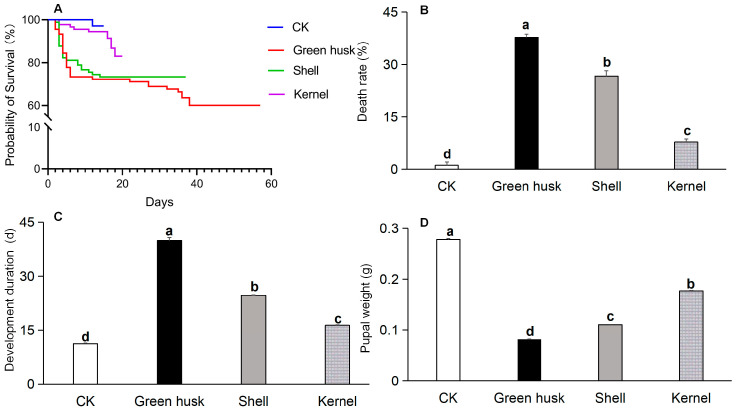
The survival rate (**A**), mortality rate (**B**), developmental duration from 2nd instar to 6th instar (**C**), and pupal weight (**D**) of *Helicoverpa armigera* fed on different parts of walnut fruits. *H. armigera* fed an artificial diet were used as the control (CK). The different letters indicate significant differences analyzed by one-way ANOVA followed by Tukey’s tests, *p* < 0.05.

Consumed food had a significant influence on development duration (*F* = 543.0, *df* = 3, 8, *p* < 0.001), mortality rate (*F* = 154.0, *df* = 3, 8, *p* < 0.001), and pupal weight (*F* = 317.4, *df* = 3, 8, *p* < 0.001). Larvae fed walnut fruit parts had developmental delays, higher mortality rates, and reduced pupal weights compared to the control group reared on artificial diets (Figure 3). Both the green husk and shell treatments led to longer developmental periods, significantly higher mortality rates, and lighter pupae compared to the kernel treatment. The green husk treatment resulted in the longest developmental period (41.5 days from the second to the sixth instars; the highest mortality rate was 37.8%) and the lightest pupae (0.08 g).

### 2.4. Larval Fresh and Dry Weights

**Figure 4:**
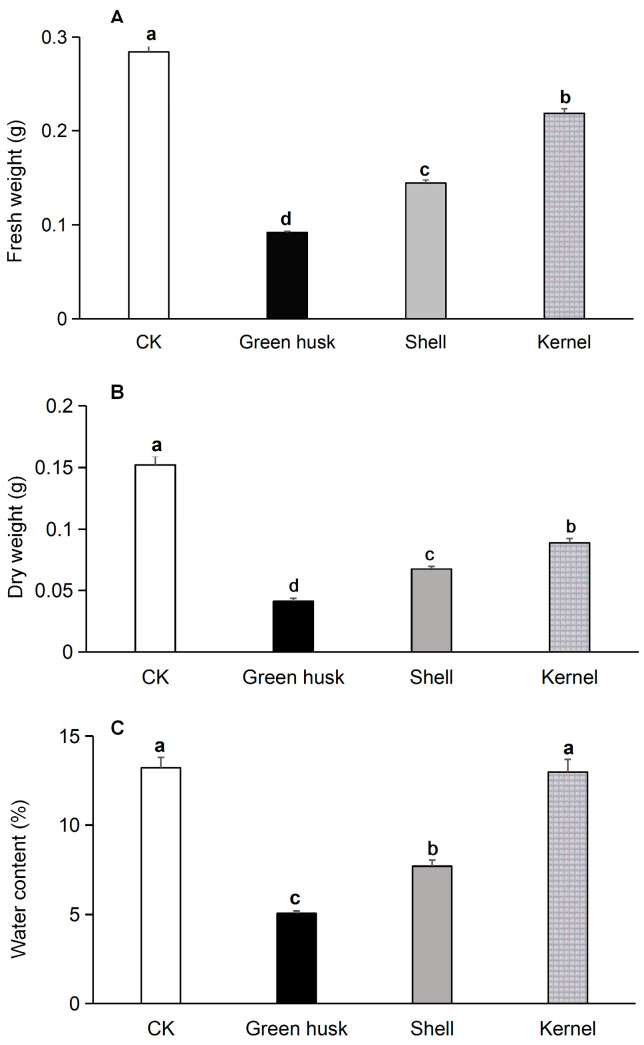
Fresh weight (**A**), dry weight (**B**), and water content (**C**) of 5th-instar larvae of *Helicoverpa armigera* fed on different parts of walnut fruits at 48 h. *H. armigera* fed an artificial diet were used as the control (CK). The different letters indicate significant differences analyzed by one-way ANOVA followed by Tukey’s tests, *p* < 0.05.

Healthy fifth instar larvae were chosen to be fed on different walnut fruit parts for 48 h, after which their fresh and dry weights were recorded (Figure 4A). Larvae fed green husks had an average fresh weight of 0.092 g, while those consuming the shell or kernel weighed 0.144 g and 0.219 g, respectively. By comparison, larvae fed artificial feed weighed 0.284 g on average. The fresh weights of larvae significantly depended on eaten food (*F* = 382.0; *df* = 3, 116, *p* < 0.001), and feeding with green husks led to significantly lower weight than all other treatments.

Average dry weight (Figure 4B) was lowest for larvae fed green husks (0.041 g), compared to 0.067 g and 0.089 g for larvae fed shells or kernels, respectively. Larvae fed on the artificial diet had the highest average dry weight (0.152 g). Dry weight was affected by food treatments (*F* = 125.9; *df* = 3, 116, *p* < 0.001) and larvae fed green husks had significantly lower dry weight than those fed other treatments or the control.

The water content of larvae fed green husks was 5.0%, compared to 8.9% for those fed shells, 13.0% fed kernels, and 15.2% fed the artificial diet (Figure 4C). Significant differences among experimental groups were recorded for water content (*F* = 65.7; *df* = 3, 116, *p* < 0.001). The water content of larvae fed with green husks was significantly lower than that of the other treatments or the control.

### 2.5. Activities of Detoxification Enzymes Depending on Treatments

**Figure 5:**
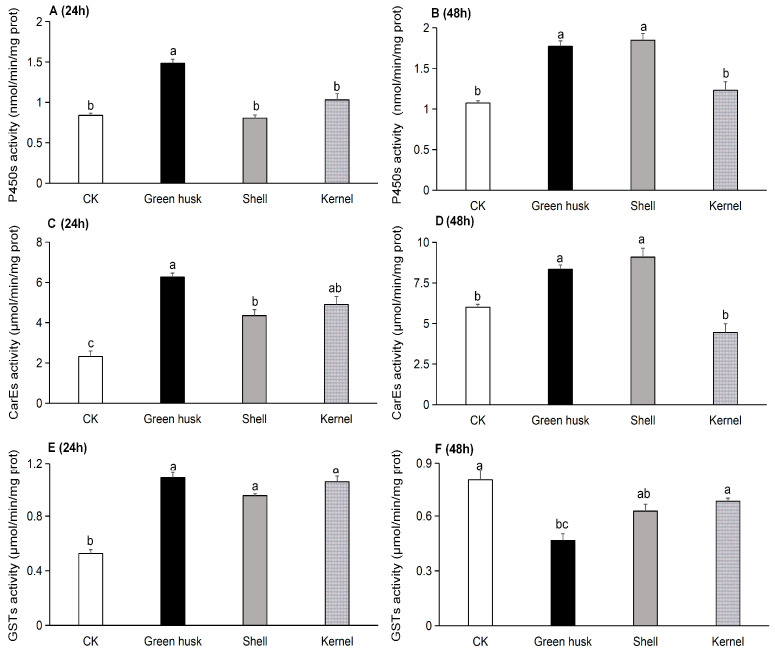
Detoxification enzyme activities of *Helicoverpa armigera* fed on different parts of walnut fruits at two different time points. The enzyme activity of P450 at 24 h (**A**) and 48 h (**B**), the activity of CarE at 24 h (**C**) and 48 h (**D**), and the activity of GST at 24 h (**E**) and 48 h (**F**). *H. armigera* fed an artificial diet were used as the control (CK). The different letters indicate significant differences analyzed by one-way ANOVA followed by Tukey’s tests, *p* < 0.05.

The activities of three major detoxifying enzymes—cytochrome P450 (P450), carboxylesterase (CarE), and glutathione S-transferase (GST) (Figure 5)—were significantly different between the various treatments of the larvae. After 24 h of feeding, the P450 enzyme activity in larvae was 1.484 nmol/min/mg prot for green husks, 0.805 nmol/min/mg prot for shells, 1.031 nmol/min/mg prot for kernels, and 0.838 nmol/min/mg prot for the artificial diet (Figure 5A). Notably, P450 activity in larvae fed green husks was significantly greater than that in larvae fed other treatments or the control (*F* = 25.1, *df* = 3, 8, *p* < 0.001). After 48 h of feeding, the P450 activities in larvae fed green husks and shells were both significantly higher than in larvae fed kernels. However, there was no significant difference in P450 enzyme activity between larvae fed green husks versus shells (Figure 5B).

The level of carboxylesterase (CarE) activity after feeding on green husks for 24 h was 6.260 µmol/min/mg prot, compared to 4.903 µmol/min/mg prot after feeding on kernels and 4.352 µmol/min/mg prot on shells. The lowest activity was 2.322 µmol/min/mg prot in the larvae fed the artificial diet. The CarE activity in the larvae fed green husks was significantly higher than those fed shells, but was not significantly different from larvae fed on kernels (Figure 5C). After 48 h, CarE activities in larvae fed green husks and shells were both significantly higher than in larvae fed kernels (Figure 5D). After 24 h, the glutathione S-transferase (GST) enzyme activity in *H. armigera* larvae that fed on different parts of the walnut fruit was significantly higher than in larvae that fed on the control (Figure 5E). After 48 h, the GST activity in larvae that were fed on green husks was significantly lower than in those fed on kernels, but was not significantly different from those that were fed on shells (Figure 5F).

### 2.6. Transcriptional Responses in Larvae Depending on Treatments

**Figure 6:**
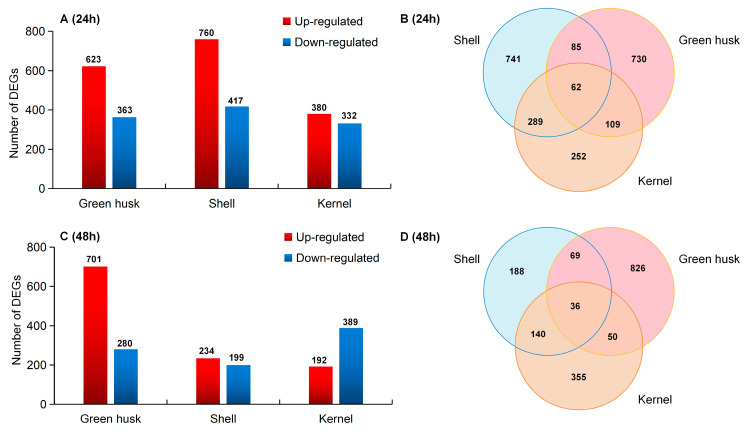
Differentially expressed genes (DEGs) in *Helicoverpa armigera* fed on different parts of walnut fruits at two different time points. Number of up- and downregulated DEGs (**A**) and Venn diagram of common and unique DEGs (**B**) at 24 h. Number of up- and downregulated DEGs (**C**) and Venn diagram of common and unique DEGs (**D**) at 48 h.

To identify the molecular mechanisms of the local adaptation of *H. armigera* to alternative host plants and to identify potential key genes and associated biological processes, transcriptomic changes in the larvae among treatments were monitored with RNA-seq analysis at 24 and 48 h of feeding. After 24 h, the numbers of DEGs in larvae fed green husks, shells, or kernels were 986 (623 up- and 363 downregulated), 1177 (760 up- and 417 downregulated), and 712 (380 up- and 332 downregulated), respectively (Figure 6A). Of these, 62 DEGs were common across all treatments (Figure 6B), while 730, 741, and 252 DEGs were specific to the green husk, shell, and kernel treatments, respectively. After 48 h of feeding, 986 (623 up- and 363 downregulated), 1177 (760 up- and 417 downregulated), and 712 (380 up- and 332 downregulated) DEGs were identified in the green husk, shell, and kernel treatments, respectively (Figure 6C). A Venn diagram showed that 36 DEGs were present in all three treatments (Figure 6D), while 826, 188, and 355 DEGs were unique to the green husk, shell, and kernel treatments, respectively.

### 2.7. Expression Patterns of DEGs in Different Treatments

**Figure 7:**
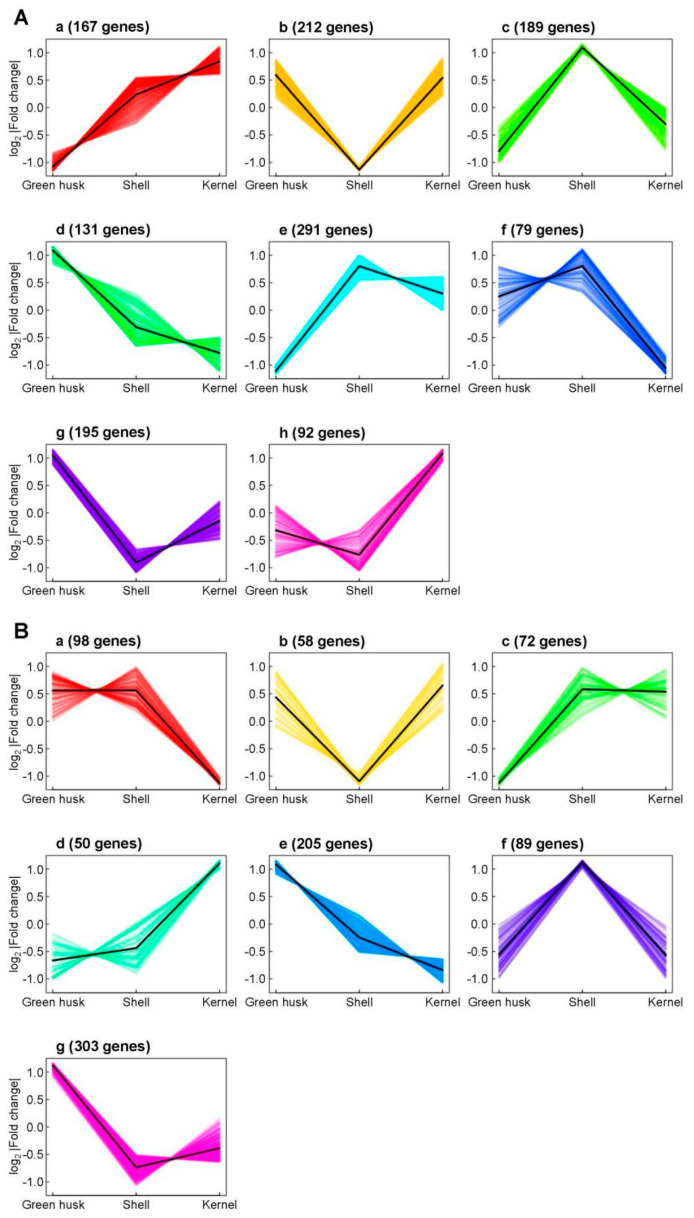
Expression profiles based on K-means with clustering of genes differentially expressed in at least one control–treatment comparison at 24 (**A**) and 48 h (**B**).

After 24 h, a total of 2268 genes were differentially expressed in at least one control-versus-treatment comparison, while 1664 genes were differentially expressed at 48 h. A k-means clustering analysis on expression patterns of these DEGs under different treatments showed distinct clusters. At 24 h, three clusters (of 647 total genes) that exhibited the lowest expression levels were associated with the green husk treatment (clusters a, c, and e). Similarly, three clusters (499 genes) that had the lowest expression levels were associated with the shell treatment (clusters b, g, and h), and two clusters (210 genes) displayed the lowest expression level for the kernel treatment (clusters d and f) (Figure 7A). At 48 h, 72 genes in one cluster that showed the lowest expression level were associated with the green husk treatment (cluster c), while 263 and 303 genes that showed the lowest expression were associated with the shell (clusters b and g) and kernel treatments (clusters a and e), respectively. In contrast, 89 genes that exhibited the highest expression were associated with the shell treatment (cluster f), and 50 genes with the highest level of expression were associated with the kernel treatment (cluster d) (Figure 7B).

### 2.8. GO and KEGG Functional Enrichment Analysis of DEGs

**Figure 8:**
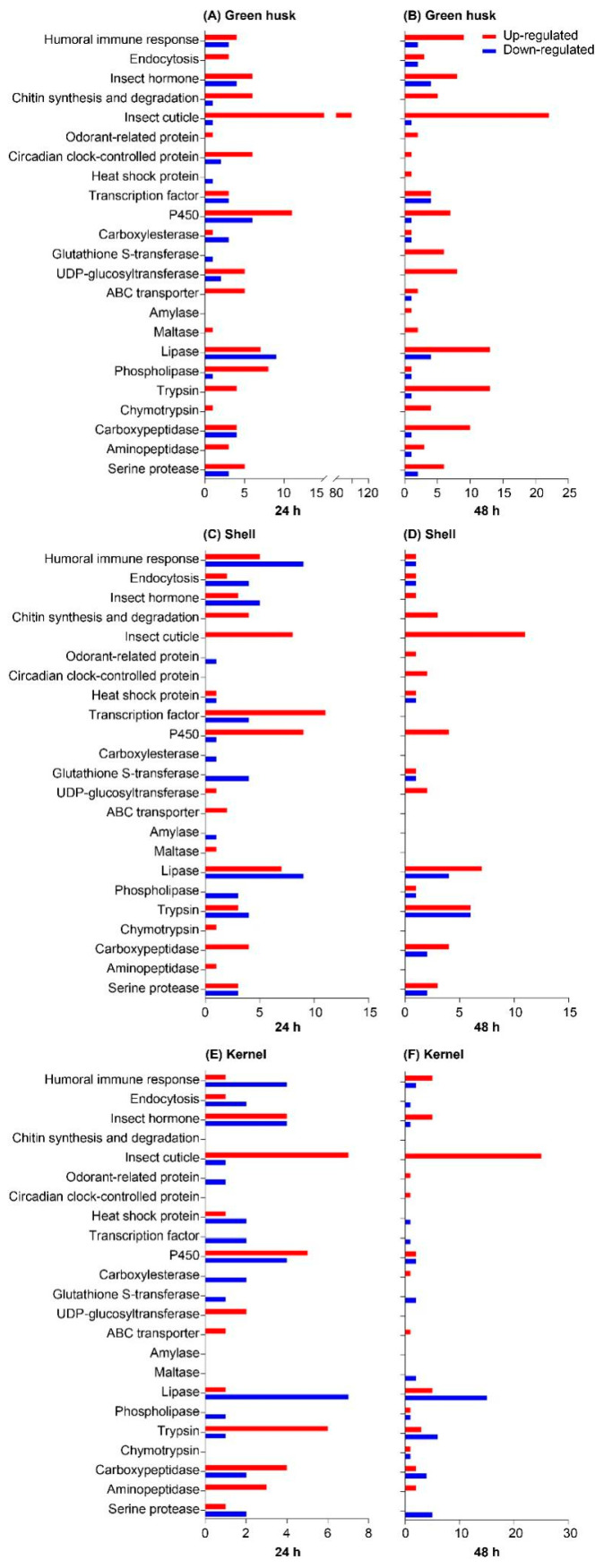
Functional classification of differentially expressed genes (DEGs) in the green husk treatment at 24 h (**A**) and 48 h (**B**), in the shell treatment at 24 h (**C**) and 48 h (**D**), and in the kernel treatment at 24 h (**E**) and 48 h (**F**).

**Figure 9:**
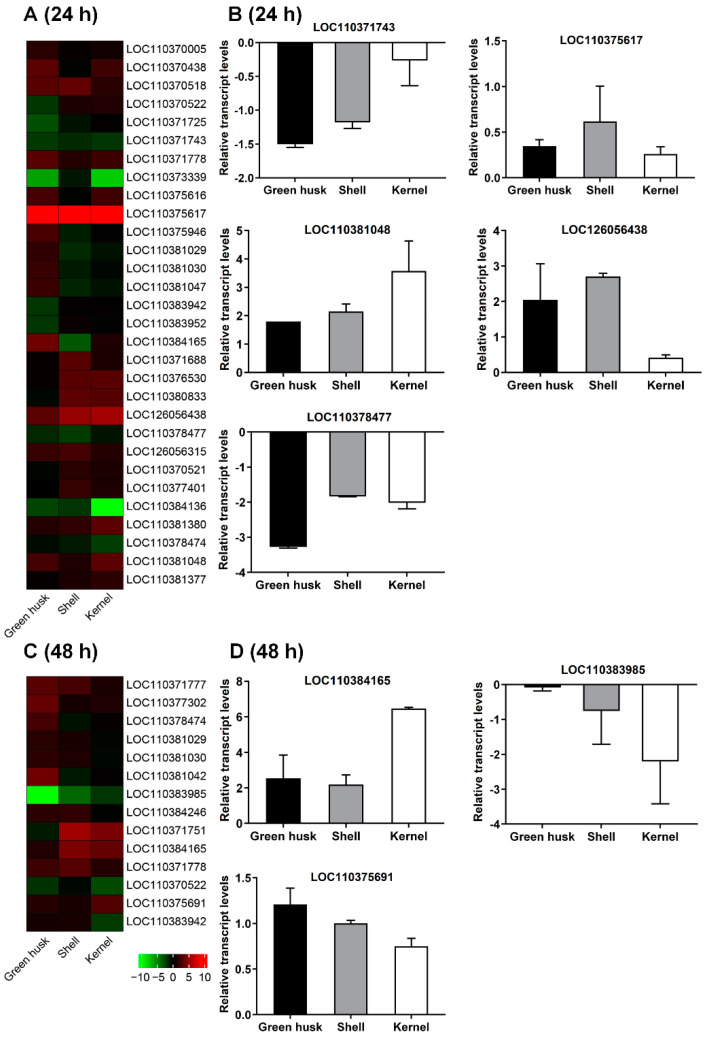
P450 genes differentially expressed in at least one control–treatment comparison in RNA-seq (**A**) and selected P450 genes in qRT-PCR (**B**) at 24 h. P450 genes differentially expressed in RNA-seq (**C**) and selected P450 genes in qRT-PCR (**D**) at 48 h. Downward-pointing bars depict downregulation and upward pointing bars depict upregulation.

GO functional enrichment and KEGG pathway analyses revealed that DEGs were mainly enriched in the following biological processes: immune response, insect hormone biosynthesis, chitin synthesis and degradation, detoxification, and digestion (Figure 8). At both 24 and 48 h, the treatments with the green husk and shell diets generally induced more DEGs encoding detoxification enzymes (P450, carboxylesterase, glutathione S-transferase, UDP-glucosyltransferase, and ABC transporter) and digestive enzymes (amylase, maltase, lipase, phospholipase, trypsin, chymotrypsin, carboxypeptidase, aminopeptidase, and serine protease) compared to the kernel treatment (Figure 8). Notably, the general induction of P450 in larvae in treatments with green husks, shells, or kernels at both time points highlights its importance and pivotal role in facilitating *H. armigera* adaptation to green walnut fruits. At 24 h, 30 P450 genes, including 12 belonging to the *CYP4* family, 12 belonging to the *CYP6* family, and 3 belonging to the *CYP9* family, were differentially expressed in at least one control-versus-treatment comparison, while 14 P450 genes showed differential expression at 48 h, with 4 from the *CYP4* family, 7 from the *CYP6* family, and 1 from the *CYP9* family (Figure 9A). In the three different treatments, the expression levels of certain genes, such as the *LOC110371778* gene, were elevated at the two time points of 24 h and 48 h. Green husk-exposed larvae exhibited the greatest number of differentially expressed P450s at both 24 h (17 genes) and 48 h (8 genes). Eight P450 genes were selected and confirmed by subsequent qPCR, and their expression levels were highly consistent with those from RNA-seq, indicating that the RNA-seq results are reliable (Figure 9B). Fourteen P450 genes were differentially expressed in RNA-seq (Figure 9C) at 48 h, including two belonging to the *CYP3* family, four belonging to the *CYP4* family, and six belonging to the *CYP6* family. In addition, two genes belonging to the *CYP9* family were differentially expressed in at least one control-versus-treatment comparison. Five P450 genes were selected and confirmed by subsequent qPCR, and their expression levels were highly consistent with those from RNA-seq, indicating that the RNA-seq results are reliable (Figure 9D).

## 3. Discussion

*Helicoverpa armigera* is a polyphagous pest known to damage several crops [16,33,34,35]. While the literature on *H. armigera* is large, few reports mention their capacity to feed on walnuts [7,10]. This study was started following field observations of *H. armigera* infestation in walnut orchards in the Xinjiang region of China. Its goal was to investigate the mechanisms by which *H. armigera* larvae were adapting to walnut fruit. In walnut production, the kernel, the edible part of the fruit, is primarily consumed for its nutritive value, while the by-products, particularly the green husk and shell, are widely used in traditional medicine and have received increasing interest in modern pharmacology due to their abundant phytochemicals and excellent antioxidant activities [36]. In this study, we first evaluated the phytochemical contents of different parts of fresh walnut fruits (green husk, shell, and kernel). Similarly to previous publications [36], our findings revealed that the green husk and shell contained substantially higher quantities of phytochemical compounds compared to the nutrient-rich kernel (Figure 1, Table S1).

We further provided *H. armigera* larvae with various parts of fresh walnut fruit (green husk, shell, and kernel) and observed their feeding preferences. Significant differences in preference for parts of the walnut fruit were noted: larvae showed the highest preference for the kernel, followed by the shell, and the green husk was the least acceptable (Figure 2). In earlier work, *H. armigera* larvae have shown a stronger affinity for cotton leaves compared to those of tobacco, tomatoes, or peppers. Significant differences in the average relative growth rates of larvae on young leaves from these four hosts were observed, and showed a descending order of cotton, tobacco, tomato, and pepper [20]. In comparison to natural substrates such as okra, tomato, eggplant, pepper, and maize, *H. armigera* exhibit a pronounced preference for ovipositing on artificial diets, with maize being the least preferred option [37,38]. In our present study, the average mortality rate of *H. armigera* larvae consuming green husks was 37.8%, followed by 26.7% for shells, 7.8% for kernels, and only 1.1% for the larvae fed on the artificial diet. The developmental duration from the second instar to the sixth instar was longest for larvae fed on green husks (41.5 days), followed by those fed on shells (25.0 days) and kernels (16.4 days), whereas only 11.2 days were needed on the artificial feed. The time required for the *H. armigera* larvae to develop on green husks was significantly longer than on the shells, kernels, or artificial diet. This pattern was also found in pupal weight. *H. armigera* larvae fed on green husks were significantly lighter than those fed on shells, kernels, or the artificial diet. Shorter developmental times, higher total reproduction, and higher pupal weight on a host plant are indicative of greater host suitability [39]. Our study similarly determined that larval development on walnut kernels was significantly shorter than on shells and green husks, with a resultant heavier pupal weight. Furthermore, larvae feeding on the green husks exhibited significantly lower average fresh and dry weights compared to those feeding on shells or kernels (Figure 4). These findings suggest that *H. armigera* larvae are better adapted to walnut kernels than green husks or shells.

Changes in gene expression in *H. armigera* larvae in the process of adapting to walnut fruits were investigated using RNA-seq analysis and subsequent qPCR validation. Functional classification revealed that the DEGs identified in this process, compared to the larvae fed the artificial diet, were primarily involved in processes essential for growth and development, such as immunity, hormone regulation, cuticle formation, detoxification, and digestion. The green husk and shell treatments induced more upregulated genes than the kernel treatment (Figure 6A,C). For example, DEGs responsible for chitin synthesis and degradation were found in the green husk and shell treatments, with almost all of these genes being upregulated; however, these DEGs were absent in the kernel treatment (Figure 7). Many DEGs responsible for cuticle protein were also identified, with almost all showing upregulation across all three treatments at both time points. Previous studies have shown that several genes encoding cuticle proteins were also induced in the midgut and fat body of *Spodoptera litura* larvae that were exposed to the botanical quinoline alkaloid compound camptothecin [40,41] and in *P. tsushimanus* larvae exposed to the plant-derived, volatile compound linalool [23].

P450 enzymes involved in the solubilization phase of detoxification play important roles in the adaptation of herbivores to the detrimental secondary metabolites of novel host plants (i.e., phytochemicals) [34,35,42,43]. For example, the P450 gene *CYP321A9* from *S. frugiperda* was found to be induced by toxic plant metabolites [44] and to be involved in the adaptation of the corn strain of *S. frugiperda* to the defensive chemicals in rice [45]. Similarly, the expression of *CYP321B1* was significantly upregulated in the midgut and fat body of *Spodoptera litura* larvae in response to tannin levels in host plants [46]. In *Mythimna separata*, RNA-seq analysis showed that seven P450 genes were significantly upregulated and the enzyme activity of *CYP450* was induced by different concentrations of chlorogenic acid, a common secondary metabolite in plants [47]. Similarly, in *H. armigera*, several P450 genes have been found to be induced by plant allelochemicals, indicating their potential role in the detoxification of these compounds. Several examples include (1) *CYP6AE14* induction in the midgut by the cotton metabolite gossypol [48], (2) *CYP6B6*, *CYP315A1*, *CYP18A1*, and *CYP306A1* induction by 2-tridecanone in wild tomato [35,49], (3) *CYP6B2*, *CYP6B6*, and *CYP6B7* induction in the midgut and fat body by four allelochemicals in many plants [50], and (4) *CYP321A1* induction by flavone in many plants [51]. In our study on walnut, the phytochemical-rich green husk and shell increased P450 enzyme activities at both time points (Figure 5A,B). Also, *H. armigera* larvae fed on the green husk, shell, or kernel treatments all upregulated multiple DEGs encoding detoxification enzymes and proteases, especially several P450s (Figure 7). Interestingly, a P450 gene (*LOC110371778*) from the *CYP4* family was upregulated in all three treatments at the 24 h and 48 h time points, suggesting it may play an important role in the detoxification of green walnut allelochemicals. This specific function requires further study. Additionally, several UDP-glucosyltransferases and ABC transporters involved in the conjunction and excretion phases of detoxification were also upregulated across all three treatments, with the green husk treatment showing the most significant gene upregulation. Similarly, both at 24 h and 48 h, CarE and GST genes were significantly upregulated in response to feeding on different parts of the walnut fruit, with the green husk treatment showing particularly significant gene upregulation.

In addition to detoxification enzymes, digestive enzymes also play a role in the adaptation of herbivorous insects to novel plants [28,52]. For example, *Hyphantria cunea* larvae exhibit varying digestive enzyme activities depending on the host plant, which plays an important role in the adaptive compensatory mechanisms of digestive enzyme inhibitors across different plants. Further studies found that the nutrients needed for the growth and development of larvae came from the activity of digestive enzymes [53]. When feeding on host plants with high nutrient content, *Heterolocha jinyinhuaphaga* larvae enhance their digestive enzyme activity to obtain more nutrients, ensuring the rapid growth of the organism [54]. These results indicate that insects adjust the activity of digestive enzymes according to their own growth and development when feeding on different host plants. In our study, feeding *H. armigera* larvae with any of the three walnut fruit treatments, at both time points, changed the expression levels of digestive enzymes (Figure 7). The number of DEGs for the green husk treatment (50 genes at 24 h and 63 at 48 h) and the shell treatment (40 at 24 h and 36 at 48 h) encoding digestive enzymes were elevated compared to the kernel treatment (28 at 24 h and 48 at 48 h) at both time points, suggesting that the green husk and shell treatments had a stronger negative influence on digestion, which in turn would affect larval growth and development. The green husk and shell treatments induced greater differential gene expression, supporting the results of the biological tests and feeding preferences. Additionally, kernel preference was more conducive to the growth and development of *H. armigera*. Many studies have demonstrated that the digestive system of insect herbivores is key to their adaptation to novel host plants during host shifts, and feeding insects new host plants with novel phytochemicals can result in the upregulation of many digestive genes [23,28,42,43,55].

## 4. Materials and Methods

### 4.1. Plant and Insect Sources

Walnut fruits of *J. regia* cv. Wen 185 were collected from the Xinjiang Experiment Station of the Chinese Academy of Agricultural Sciences, Aksu, Xinjiang, China (80.34° E, 41.12° N). The adults of *Helicoverpa armigera* collected from the Xinjiang Experiment Station were paired for matin; the resulting offspring larvae were subsequently reared on an artificial diet under controlled conditions (25 ± 1 °C, 60 ± 5% RH, and a 16:8 L:D photoperiod) [56].

### 4.2. Determination of Secondary Metabolite Content

Samples of 2 g were extracted with 60% acetone and measured using the Folin–Ciocalteu method as described previously [57], with some modifications. The absorbance was measured at 725 nm. The total alkaloid assay was performed with berberine as a reference standard, as reported previously [10]. Total polyphenols were measured by the Folin–Ciocalteu method as previously reported [58], with some modifications. Briefly, 1 g samples were extracted by 60% ethanol. The absorbance was measured at 778 nm. The gallic acid solution was used as a standard. Total flavonoid content was determined using aluminum chloride reagent as described previously [59]. The absorbance was measured at 415 nm. The rutin standard solution was used as a reference standard. Monomeric phenolics including gallic acid, chlorogenic acid, and p-hydroxybenzoic acid (PHBA) were measured on an Agilent 1220 Infinity II gradient liquid chromatography system equipped with an Agilent ZORBAX SB-C18 column (Santa Clara, CA, USA). A total of 2.0 g of the sample was extracted by 75% methanol. Solvent A: 0.1% formic acid; solvent B: acetonitrile. Gradient elution was as follows: 0–5 min, 5% to 20%; 5–20 min, 20% to 100%; 20–21 min, 100% to 5%; 21–30 min, 5%. The flow rate was 0.5 mL/min. An injection volume of 10 µL was used. These metabolites were verified by the synthesized standards.

### 4.3. Larval Feeding Preferences on Walnut Fruit 

Walnut fruit parts (green husk, shell, and kernel) were taken as the initial area, then cut into slices with a thickness of 2 mm with a knife. In each Petri dish, a thin slice of walnut was placed alongside a 3rd-instar larva of *H. armigera*. Image J software (v1.8.0) was used to measure the area consumed by the larvae before and after a 24 h period of 3rd-instar feeding to determine the relative preferences of larvae among parts. The average feeding area of *H. armigera* from 30 Petri dishes was used as one replicate, and this was repeated three times, for a total of 90 Petri dishes. The experimental conditions were 25 ± 1 °C and 60 ± 5% RH, and a 16:8 L:D photoperiod.

### 4.4. Determination of Fresh and Dry Weights of Test Larvae

To establish breeding protocols for *H. armigera*, we selected newly molted 5th-instar and placed each larva individually in sterile vials. Each larva was fed either green husk, shell, or kernel tissue, with an artificial diet serving as the control. After 48 h, we recorded the fresh weights of 30 larvae from each treatment. The larvae were subsequently dried at 80 °C until a constant weight was reached to determine their dry weights. The water content of the larvae was calculated by subtracting the dry weight from the fresh weight [60].

### 4.5. Growth and Development, Pupal Weight

The growth and development of *H. armigera* larvae were evaluated by providing them with green husk, shell, or kernel diets. Initially, neonate larvae were supplied with a specialized artificial diet tailored for the nutritional requirements of *H. armigera*. Upon entering the second instar, the larvae were placed individually in tubes and provided with one of the three walnut fruit parts. There were 30 larvae in each treatment, and the whole experiment was replicated three times, for 90 larvae per treatment. The experimental conditions were 25 ± 1 °C, 60 ± 5% RH, and a 16:8 L:D photoperiod. Fresh walnut material was supplied daily, and both the survival and developmental stages of the larvae were recorded daily. Pupal weights were measured upon larval pupation.

### 4.6. Detoxification Enzyme Activities

To assess detoxification enzymes’ activities, *H. armigera* larvae were reared to the 3rd instar on an artificial diet. At the conclusion of this stage, larvae were fed green husks, shells, or kernels as treatments and an artificial diet as the control. We then examined the impact of these treatments 24 and 48 h after the exposure to the diets by measuring the levels of the primary detoxification enzymes. Larvae were collected at random within treatments, and whole-body tissue was collected. Twenty individuals were used per treatment, and each treatment was repeated three times, for a total of sixty larvae. Protein concentrations were measured using the TP Assay Kit (A045-2) sourced from Nanjing Jiancheng Bioengineering Institute (Nanjing, China). The activities of three key detoxification enzymes—carboxylesterase (CarE), Glutathione S—transferase (GST), and cytochrome P450 (P450)—were determined using the Carboxylesterase Assay Kit (A133-1-1), Glutathione S—transferase (GSH-ST) Assay kit(A004-1-1), and Cytochrome P450 Assay Kit (H303-1-2), respectively, adhering to the protocols provided by the Nanjing Jiancheng Bioengineering Institute (Nanjing, China).

### 4.7. Total RNA Extraction and Transcriptome Sequencing

Larvae of consistent size at the 3rd instar were selected and fed different walnut fruit parts (green husk, shell and kernel). These larvae were then collected 24 or 48 h after the start of the treatment. Control larvae were reared on an artificial diet. Each treatment contained 30 larvae and the whole experiment was replicated three times (so, 90 [3 × 30] larvae were tested for each treatment). Subsequently, total RNA from the sampled larvae was isolated using the TRIzol reagent (Invitrogen Life Technologies, Carlsbad, CA, USA) and subjected to RNA sequencing analysis. RNA concentration and purity were assessed using a NanoDrop spectrophotometer (Thermo Scientific, Wilmington, NC, USA). For cDNA library preparation, 3 μg of total RNA per sample was used as the input material with the NEBNext Ultra II RNA Library Prep Kit for Illumina according to the manufacturer’s instructions. Sample sequencing was conducted on the Illumina NovaSeq 6000 platform (Personal Biotechnology Co., Ltd., Shanghai, China). Clean reads were aligned to the *H. armigera* reference genome available at NCBI (https://www.ncbi.nlm.nih.gov/datasets/genome/GCF_023701775.1, accessed on 26 October 2023) using HISAT2 (v2.1.0). Genes with |log2FoldChange| > 1 and *p* < 0.05 were identified as differentially expressed genes (DEGs). GO enrichment analysis and KEGG pathway enrichment analyses were performed using the topGO (v2.50.0) and ClusterProfiler (v4.6.0) packages, respectively.

### 4.8. qPCR Validation

Total RNA used for transcriptome sequencing was reverse-transcribed into cDNA using the PrimeScript™ 1st Strand cDNA Synthesis Kit (TaKaRa, Shiga, Japan). The qPCR validation was performed using the AceQ^®^ qPCR SYBR^®^ Green Master Mix kit (Vazyme Biotech, Nanjing, China) following the manufacturer’s protocols [61]. The qPCR amplification conditions were 95 °C for 5 min, followed by 40 cycles of 95 °C for 15 s and 60 °C for 30 s. The actin gene of *H. armigera* was used as a candidate reference gene [62]. All primers used for qPCR are listed in Table S3.

### 4.9. Statistical Analysis

Principal component analysis (PCA) was used to indicate the distribution of secondary metabolites in various tissues of the walnut fruit—specifically, the green husk, shell, and kernel. PCA was performed by using the “vegan” package [63] in R 4.2.1 software [64]. All values shown are means ± SE (standard errors). Significant differences for feeding preference, fresh weight, dry weight, growth, development, pupal weight, and detoxification enzyme activities between larvae fed walnut fruit parts and the artificial diet were analyzed using one-way ANOVA, followed by Tukey’s tests for mean separation. Statistical difference was considered significant at *p* < 0.05.

## 5. Conclusions

Crop diversity in the Aksu region of Xinjiang has increased the exposure of alternate, marginal, or poor-quality crops such as walnut to *H. amigera*. In this study, we provide insights into the interaction between *H. armigera* and this alternate host. Different parts of the walnut fruit exert distinct effects on the feeding preferences, larval growth and development, survival rates, and enzyme activity levels of larvae, with the phytochemical-rich green husk and shell having greater negative impacts compared to the nutrient-rich kernel. RNA-seq analysis showed that feeding on different walnut parts strongly affected the expression of genes involved in detoxification, digestion, immunity, and cuticle formation. More P450 genes were upregulated in the green husk and shell treatments than in the kernel treatment. These findings improve our understanding of the adaptive mechanisms being used by *H. amigera* to feed on walnut fruits specifically, and, more generally, also contribute to our understanding of host adaptation in phytophagous insects to alternate, marginal, or poor-quality hosts.

Finally, considerably more work will need to be carried out to determine the molecular functions of the P450 candidate genes and other genes involved in detoxification metabolism and their co-expression relationship.

## Data Availability

Data are contained within the article or Supplementary Materials.

## Supplementary Materials

The following supporting information can be downloaded at: https://www.mdpi.com/article/10.3390/plants13192761/s1, Table S1: Content of secondary metabolites in different parts of walnut fruit; Table S2: Duration of larval instars (means ± SE) from the second (2L) to the sixth (6L) expressed in days for *Helicoverpa armigera* larvae fed on different walnut parts; Table S3: Primers used for qRT-PCR analysis in this study.

## References

[1] Fitt G.P. (1989). The ecology of *Heliothis* species in relation to agroecosystem. Annu. Rev. Entomol..

[2] Smith C.W. (1992). History and Status of Host Plant Resistance in Cotton to Insects in the United States. Adv. Agron..

[3] Naseri B., Fathipour Y., Moharramipour S., Hosseininaveh V. (2010). Nutritional indices of the cotton bollworm, *Helicoverpa armigera*, on 13 soybean varieties. J. Insect Sci..

[4] Hemati S.A., Naseri B., Ganbalani G.N., Dastjerdi H.R., Golizadeh A. (2012). Effect of different host plants on nutritional indices of the pod borer, *Helicoverpa armigera*. J. Insect Sci..

[5] Tay W.T., Soria M.F., Walsh T., Thomazoni D., Silvie P., Behere G.T., Anderson C., Downes S. (2017). A brave new world for an old world pest: *Helicoverpa armigera* (Lepidoptera: Noctuidae) in Brazil. PLoS ONE.

[6] Cunningham J.P., Zalucki M.P. (2014). Understanding heliothine (Lepidoptera: Heliothinae) pests: What is a host plant?. J. Econ. Entomol..

[7] Yang L., Liu H., Pan Y., Li H., Lu Y. (2022). Landscape simplification increases the risk of infestation by the polyphagous pest *Helicoverpa armigera* for walnut, a novel marginal host. Landsc. Ecol..

[8] Wu K.M., Guo Y.Y. (2005). The evolution of cotton pest management practices in China. Annu. Rev. Entomol..

[9] Li H.Q., Li J.H., Yang L., Liu J., Lu Y.H. (2017). Preliminary report on the investigation of the damage of *Helicoverpa armigera* to walnut. China Plant Prot..

[10] Liu H., Xiu C., Zhang T., Lu Y. (2022). Odor perception in the cotton bollworm, *Helicoverpa armigera*, exposed to *Juglans regia*, a marginal host plant. J. Chem. Ecol..

[11] Ntalli N., Koliopoulos G., Giatropoulos A., Menkissoglu-Spiroudi U. (2019). Plant secondary metabolites against arthropods of medical importance. Phytochem. Rev..

[12] Magalhaes D.M., Borges M., Laumann R.A., Caulfield J.C., Blassioli-Moraes M.C. (2020). Inefficient weapon-the role of plant secondary metabolites in cotton defense against the boll weevil. Planta.

[13] Maurya A.K., Patel R.C., Frost C.J. (2020). Acute toxicity of the plant volatile indole depends on herbivore specialization. J. Pest Sci..

[14] Wari D., Aboshi T., Shinya T., Galis I. (2022). Integrated view of plant metabolic defense with particular focus on chewing herbivores. J. Integr. Plant Biol..

[15] Sarfraz M., Dosdall L.M., Keddie B.A. (2006). Diamondback moth-host plant interactions: Implications for pest management. Crop Prot..

[16] Hemati S.A., Naseri B., Razmjou J. (2013). Reproductive performance and growth indices of the cotton bollworm, *Helicoverpa armigera* (Lepidoptera: Noctuidae) on various host plants. Crop Prot..

[17] Heckel D.G. (2014). Insect detoxifcation and sequestration strategies. Annu. Rev. Plant Biol..

[18] Heidel-Fischer H.M., Vogel H. (2015). Molecular mechanisms of insect adaptation to plant secondary compounds. Curr. Opin. Insect Sci..

[19] Petschenka G., Agrawal A.A. (2016). How herbivores coopt plant defenses: Natural selection, specialization, and sequestration. Curr. Opin. Insect Sci..

[20] Ruan Y.M., Wu K.J. (2001). Effects of different feeding plants on the growth, development and reproduction of cotton bollworm. Acta Entomol. Sin..

[21] Schellhorn N.A., Pierce S., Bianchi F.J.J.A., Williams D., Zalucki M.P. (2008). Designing landscapes for multiple outcomes in broadacre environments. Aust. J. Exp. Agric..

[22] Razmjou J., Naseri B., Hemati S.A. (2014). Comparative performance of the cotton bollworm, *Helicoverpa armigera* (Hübner) (Lepidoptera: Noctuidae) on various host plants. J. Pest Sci..

[23] Li S., Li H., Chen C., Hao D. (2023). Tolerance to dietary linalool primarily involves co-expression of cytochrome P450s and cuticular proteins in *Pagiophloeus tsushimanus* (Coleoptera: Curculionidae) larvae using SMRT sequencing and RNA-seq. BMC Genom..

[24] Jin M., Liao C., Fu M.X., Holdbrook R., Wu K., Xiao Y. (2019). Adaptive regulation of detoxification enzymes in *Helicoverpa armigera* to different host plants. Insect Mol. Biol..

[25] Robertson H.M., Martos R., Sears C.R., Todres E.Z., Walden K.K.O., Nardi J.B. (1999). Diversity of odourant binding proteins revealed by an expressed sequence tag project on male *Manduca sexta* moth antennae. Insect Mol. Biol..

[26] Crava C.M., Brutting C., Baldwin I.T. (2016). Transcriptome profiling reveals differential gene expression of detoxification enzymes in a hemimetabolous tobacco pest after feeding on jasmonate-silenced *Nicotiana attenuata* plants. BMC Genom..

[27] Shi Y., Qu Q., Wang C., He Y., Yang Y., Wu Y. (2022). Involvement of *CYP2* and mitochondrial clan P450s of *Helicoverpa armigera* in xenobiotic metabolism. Insect Mol. Biol..

[28] Jeckel A.M., Beran F., Züst T., Younkin G., Petschenka G., Pokharel P., Robert C.A.M. (2022). Metabolization and sequestration of plant specialized metabolites in insect herbivores: Current and emerging approaches. Front. Physiol..

[29] Heiko V., Richard O.M., Mariam P.C.M. (2014). Transcriptome responses in herbivorous insects towards host plant and toxin feeding. Annu. Rev. Plant Biol..

[30] Coates B.S., Walden K.K., Lata D., Vellichirammal N.N., Mitchell R.F., Andersson M.N., Robertson H.M. (2023). A draft *Diabrotica virgifera* genome: Insights into control and host plant adaption by a major maize pest insect. BMC Genom..

[31] Birnbaum S.S., Abbot P. (2020). Gene expression and diet breadth in plant-feeding insects: Summarizing trends. Trends Ecol. Evol..

[32] Pym A., Troczka B.J., Hayward A., Zeng B., Gao C.F., Elias J., Bass C. (2024). The role of the *Bemisia tabaci* and *Trialeurodes vaporariorum* cytochrome-P450 clade *CYP6DPx* in resistance to nicotine and neonicotinoids. Pestic. Biochem. Physiol..

[33] Hemati S.A., Naseri B., Ganbalani G.N., Dastjerdi H.R., Golizadeh A. (2012). Digestive proteolytic and amylolytic activities and feeding responses of *Helicoverpa armigera* (Noctuidae: Lepidoptera) on different host plants. J. Econ. Entomol..

[34] Song C., Yihua Y., Yidong W. (2005). Correlation between fenvalerate resistance and cytochrome P450-mediated o-demethylation activity in *Helicoverpa armigera* (Lepidoptera: Noctuidae). J. Econ. Entomol..

[35] Zhang L., Lu Y., Xiang M., Shang Q., Gao X. (2016). The retardant effect of 2-tridecanone, mediated by cytochrome P450, on the development of cotton bollworm, *Helicoverpa armigera*. BMC Genom..

[36] Jahanban-Esfahlan A., Ostadrahimi A., Tabibiazar M., Amarowicz R. (2019). A comprehensive review on the chemical constituents and functional uses of walnut (*Juglans* spp.) husk. J. Mol. Sci..

[37] Jallow M.F.A., Matsumura M., Suzuki Y. (2001). Oviposition preference and reproductive performance of Japanese *Helicoverpa armigera* (Hübner) (Lepidoptera: Noctuidae). Appl. Entomol. Zool..

[38] Jallow M.F., Zalucki M.P. (2003). Relationship between oviposition preference and offspring performance in Australian *Helicoverpa armigera* (Hübner) (Lepidoptera: Noctuidae). Aust. J. Entomol..

[39] Van Lenteren J.C., Noldus L.P.J.J., Gerling D. (1990). Whitefly-plant relationship: Behavioral and biological aspects. Whitefly: Their Bionomics, Pest Management Science.

[40] Shu B., Zou Y., Yu H., Zhang W., Li X., Cao L., Lin J. (2021). Growth inhibition of *Spodoptera frugiperda* larvae by camptothecin correlates with alteration of the structures and gene expression profiles of the midgut. BMC Genom..

[41] Shu B., Yang X., Dai J., Yu H., Yu J., Li X., Lin J. (2021). Effects of camptothecin on histological structures and gene expression profiles of fat bodies in *Spodoptera frugiperda*. Ecotox. Environ. Saf..

[42] Lu K., Song Y., Zeng R. (2021). The role of cytochrome P450-mediated detoxification in insect adaptation to xenobiotics. Curr. Opin. Insect Sci..

[43] Li S.Y., Li H., Wang J.T., Chen C., Hao D.J. (2023). Hormetic response and co-expression of cytochrome P450 and cuticular protein reveal the tolerance to host-specific terpenoid defenses in an emerging insect pest, *Pagiophloeus tsushimanus* (Coleoptera: Curculionidae). J. Pest Sci..

[44] Giraudo M., Hilliou F., Fricaux T., Audant P., Feyereisen R. (2015). Cytochrome P450s from the fall armyworm (*Spodoptera frugiperda*): Responses to plant allelochemicals and pesticides. Insect Mol. Biol..

[45] He L., Shi Y., Ding W.B., Huang H., He H.L., Xue J., Gao Q., Zhang Z.X., Li Y.Z., Qiu L. (2023). Cytochrome P450s genes *CYP321A9* and *CYP9A58* contribute to host plant adaptation in the fall armyworm *Spodoptera frugiperda*. Pest Manag. Sci..

[46] Zhao P., Xue H., Zhu X., Wang L., Zhang K., Li D., Ji J., Niu L., Gao X., Luo J. (2022). Silencing of cytochrome P450 gene *CYP321A1* effects tannin detoxification and metabolism in *Spodoptera litura*. Int. J. Biol. Macromol. Struct. Funct. Interact..

[47] Lin D., Fang Y., Li L., Zhang L., Gao S., Wang R., Wang J. (2022). The insecticidal effect of the botanical insecticide chlorogenic acid on *Mythimna separata* (Walker) is related to changes in *MsCYP450* gene expression. Front. Plant Sci..

[48] Mao Y.B., Cai W.J., Wang J.W., Hong G.J., Tao X.Y., Wang L.J., Chen X.Y. (2007). Silencing a cotton bollworm P450 monooxygenase gene by plant-mediated RNAi impairs larval tolerance of gossypol. Nat. Biotechnol..

[49] Liu X.N., Liang P., Gao X.W., Shi X.Y. (2006). Induction of the cytochrome P450 activity by plant allelochemicals in the cotton bollworm, *Helicoverpa armigera* (Hübner). Pestic. Biochem. Physiol..

[50] Chen S., Elzaki M.E.A., Ding C., Li Z.F., Wang J., Zeng R.S., Song Y.Y. (2019). Plant allelochemicals affect tolerance of polyphagous lepidopteran pest *Helicoverpa armigera* (Hübner) against insecticides. Pestic. Biochem. Physiol..

[51] Zhang C., Wang X., Tai S., Qi L., Yu X., Dai W. (2023). Transcription factor CncC potentially regulates cytochrome P450 *CYP321A1*-mediated flavone tolerance in *Helicoverpa armigera*. Pest. Biochem. Physiol..

[52] Simon J.C., d’Alencon E., Guy E., Jacquin-Joly E., Jaquiery J., Nouhaud P., Streiff R. (2015). Genomics of adaptation to host plants in herbivorous insects. Insect Mol. Biol..

[53] Zhang A., Li T., Yuan D.Y.S. (2023). Digestive characteristics of *Hyphantria cunea* larvae on different host plants. Insects.

[54] Xiang Y.Y., Sun X., Yin P.F. (2020). Effects of host plants and temperatures on digestive enzyme activities in *Heterolocha jinyinhuaphaga* larvae. J. Zhejiang AF Univ..

[55] Roy A., Walker W.B., Vogel H., Chattington S., Larsson M.C., Anderson P., Schlyter F. (2016). Diet dependent metabolic responses in three generalist insect herbivores *Spodoptera* spp. Insect Biochem. Mol. Biol..

[56] Liang G.M., Wu K.M., Yu H.K., Li K.K., Feng X., Guo Y.Y. (2008). Changes of inheritance mode and fitness in *Helicoverpa armigera* (Hübner) (Lepidoptera: Noctuidae) along with its resistance evolution to Cry1Ac toxin. J. Invertebr. Pathol..

[57] Nyero A., Anywar G.U., Achaye I., Malinga G.M. (2023). Phytochemical composition and antioxidant activities of some wild edible plants locally consumed by rural communities in northern Uganda. Front. Nutr..

[58] Złotek U., Świeca M., Jakubczyk A. (2014). Effect of abiotic elicitation on main health-promoting compounds, antioxidant activity and commercial quality of butter lettuce (*Lactuca sativa* L.). Food Chem..

[59] Yang J., Ma C., Jia R., Zhang H., Zhao Y., Yue H., Jiang X. (2023). Different responses of two maize cultivars to *Spodoptera frugiperda* (Lepidoptera: Noctuidae) larvae infestation provide insights into their differences in resistance. Front. Plant Sci..

[60] Jia Y.L., Cheng X.D., Cai Y.P., Luo M.H., Guo X.R., Yuan G.H. (2012). Host fitness of cotton bollworm in four pepper varieties. Acta Ecol. Sin..

[61] Huang Y., Zheng J.Y., Wu P.Z., Zhang Y.Z., Qiu L.H. (2023). A comparative study of transcriptional regulation mechanism of cytochrome P450 *Cyp6b7* between resistant and susceptible strains of *Helicoverpa armigera*. J. Agric. Food Chem..

[62] Su C.Y., Liu S.S., Sun M.X., Yu Q.L., Li C.Y., Graham R.I., Wang X.F., Wang X.W., Xu P.J., Ren G.W. (2023). Delivery of methoprene-tolerant dsRNA to improve RNAi efficiency by modified liposomes for pest control. ACS Appl. Mater. Interfaces.

[63] Oksanen J., Simpson G., Blanchet F., Kindt R., Legendre P., Minchin P. (2022). Vegan: Community Ecology Package, R package version 2.6-2.

[64] R Development Core Team (2022). R: A Language and Environment for Statistical Computing.

