# Supplementary material for: Behavioral, Physiological, and Molecular Mechanisms Underlying the Adaptation of Helicoverpa armigera to the Fruits of a Marginal Host: Walnut (Juglans regia)
Source: Plants (Basel). 2024 Oct 1;13(19):2761. doi: 10.3390/plants13192761 (PMC11478790; doi:10.3390/plants13192761)
Supplement: Supplementary file 1 [file plants-13-02761-s001.zip › plants-3199332-supplementary.pdf]

plants

MDPI

*Supplementary Data*

# Behavioral, physiological and molecular mechanisms underlying the adaptation of *Helicoverpa armigera* to fruits of a marginal host, walnut (*Juglans regia*)

**Haiqiang Li [1,2], Xinzheng Huang[3] , Long Yang[1], Haining Liu[1], Bing Liu[1] and Yanhui Lu[1] ***

**Table S1.** Content of secondary metabolites in different parts of walnut fruit.

| Walnut fruit | Tannin (mg/kg ) | Total alkaloids(% ) | Total polyphenol (mg/kg ) | Total flavone (mg/kg ) | Gallic acid (mg/kg ) | Chlorogenic acid (mg/kg ) | p-Hydroxybenzoic acid (PHBA) (mg/kg ) |
|---|---|---|---|---|---|---|---|
| Green husk | 7588.67±63.613b | 0.73±0.004b | 8.64±0.035b | 2833.33±72.001a | 153.10±0.205a | 30.37±0.250 b | 9.64±0.135b |
| Shell | 13296.67±273.103a | 0.88±0.020a | 13.09±0.075a | 1333.33±27.217b | 19.29±0.357c | 163.87±1.992 a | 10.72±0.254a |
| Kernel | 2181.00±49.108c | 0.22±0.001c | 1.79±0.036c | 1123.33±62.600b | 24.78±0.305b | 11.57±0.179c | 5.54±0.068c |

Note: Data are Mean ± SE; the same lowercase letters within columns indicate no significant difference based on ANOVA followed by Tukey's MRT, $\alpha = 0.05$.

plants MDPI

**Table S2.** Duration of larval instars (means ± SE) from the second (2L) to the sixth (6L) expressed in days for *Helicoverpa armigera* larvae fed on different walnut parts

| Feeding food | 2L | 3L | 4L | 5L | 6L |
|---|---|---|---|---|---|
| CK (artificial diet) | 1.73±0.057 | 2.26±0.074 | 2.47±0.096 | 2.43±0.063 | 2.36±0.031 |
| Green husk | 6.16±0.151 | 7.96±0.739 | 6.92±0.205 | 8.56±0.055 | 11.49±0.516 |
| Shell | 3.58±0.018 | 6.02±0.056 | 5.77±0.123 | 5.78±0.412 | 4.84±0.123 |
| Kernel | 3.16±0.089 | 3.54±0.055 | 3.28±0.078 | 4.26±0.285 | 2.86±0.074 |

plants MDPI

**Table S3.** Primers used for qRT-PCR analysis in this study.

| Name | Sequence (5′→3′) |
| --- | --- |
| LOC110375617-F | AGAACACTACTGGCCAGCAA |
| LOC110375617-R | AGAACACTACTGGCCAGCAA |
| LOC110371743-F | AACCTCTTCTCCACTTCCGG |
| LOC110371743-R | CTTCGCGGAGGTAAACACAG |
| LOC126056438-F | TGGTGTTGGGAGTCCTGATC |
| LOC126056438-R | AACCAGGACCATATCGCCAA |
| LOC110381048-F | GGGGCTATGAGAAGGAGGTC |
| LOC110381048-R | AGTGTCGAAGCCTCCAAGAA |
| LOC110378477-F | CCTGTTCTTTGCTGCTGGTT |
| LOC110378477-R | ACTTGCCACCACTCTTAGCA |
| LOC110384165-F | CGCTCGTCGGTGATACTG |
| LOC110384165-R | AACCTCTGATTGTGCCTTCTT |
| LOC110375691-F | ACTCGGAAATCAGCTGACGA |
| LOC110375691-R | CGCTGTTGATGATGCCAAGA |
| LOC110383985-F | CAGTTTGTCCTGGCAATGCT |
| LOC110383985-R | TTTGTCCTGAATCTCCGGCT |
| actin(nei)-F | GTATGGAATCCTGCGGTAT |
| actin(nei)-R | GGTCCTTACGGATGTCAA |

Fwd: forward primer; Rev: reverse primer; *sfRpl-10: ribosomal protein L10.*
